# *Terminalia Arjuna* bark extract impedes foam cell formation and promotes apoptosis in ox-LDL-stimulated macrophages by enhancing UPR-CHOP pathway

**DOI:** 10.1186/s12944-019-1119-z

**Published:** 2019-11-10

**Authors:** Shipra Bhansali, Shivani Khatri, Veena Dhawan

**Affiliations:** Department of Experimental Medicine and Biotechnology, Research Block-B, Postgraduate Institute of Medical Education and Research (PGIMER), Chandigarh, 160012 India

**Keywords:** Atherosclerosis, Macrophages, Foam cells, *Terminalia Arjuna* bark extract, JNK and p38MAPK signaling, Unfolded protein response (UPR) pathway, Apoptosis

## Abstract

**Background:**

Increased macrophage and foam cell apoptosis during early atherogenesis retards plaque progression by impeding foam cell formation, suppressing inflammation and limiting lesion cellularity. Our previous in vitro study in THP1 macrophages demonstrated that *Terminalia Arjuna* (TA) attenuates dual-specificity phosphatase1 (DUSP1), a key negative regulator of JNK/P38MAPK signaling cascade, the branch also implicated in the UPR (unfolded protein response)-CHOP-mediated apoptotic pathway; however this pathway has not been explored so far in the presence of TA. Therefore, we aimed to elucidate the pro-apoptotic effect of aqueous bark extract of TA (aqTAE) on macrophage and foam cells and the underlying mechanism associated with it.

**Methods:**

THP1 cells were initially differentiated into macrophages with phorbol-12-myristate-13-acetate (PMA) (100 ng/ml) for 24 h, followed by ox-LDL (100 μg/ml) treatment for another 24 h to induce foam cell formation. Thereafter, macrophages and ox-LDL- treated cells were incubated with aqTAE (100 μg/ml) for the next 24 h. Further, Oil Red O (ORO) staining, CD36 expression profiling, apoptotic assay and transcriptional and translational expression of ER-stress markers i.e., X-box binding protein 1 (XBP1) and C/EBP homologous protein (CHOP) were performed for elucidating the potential mechanism underlying TA-induced macrophage and foam cell apoptosis.

**Results:**

We demonstrated that ox-LDL treatment significantly increased lipid accumulation and upregulated CD36 expression, indicating foam cell formation; while the addition of aqTAE resulted in a significant decline in ORO positive cells, and suppression of CD36 expression in ox-LDL-stimulated macrophages, suggestive of reduced formation of lipid-laden foam cells. Further, aqTAE treatment alone and in combination with oxidized low-density lipoprotein (ox-LDL) stimulus, significantly attenuated CD36 expression; increased apoptosis; and augmented the expression of UPR regulatory proteins including XBP1 and CHOP, and similar observations were noted when cells were treated with ox-LDL alone. These findings indicate that TA promotes macrophage and foam cell apoptosis via enhancing UPR-mediated activation of JNK/p38MAPK-CHOP pathway in a DUSP1-dependent manner, implying a possible interplay between ox-LDL-induced ER stress- and TA-mediated MAPK signaling.

**Conclusion:**

Our data shows that aqTAE inhibits foam cell formation, as well as promotes macrophage and foam cell apoptosis by augmenting UPR- JNK/p38MAPK-CHOP signaling cascade via inhibiting DUSP1. These findings provide novel mechanistic insight into the anti-atherogenic potential of TA, which may prove beneficial against early-stage atherosclerotic lesions.

## Introduction

Atherosclerosis is considered as a chronic inflammatory disease of the arterial wall, characterized by the formation of atherosclerotic plaque in the sub-endothelial space that comprises of migrated smooth muscle cells (SMCs), oxidized lipids, apoptotic macrophages, foam cells, activated leukocytes, and inflammatory cytokines [1]. Macrophages play a pivotal role in atherosclerosis, as they are the primary cells to invade atherosclerotic lesions and ingest oxidized low-density lipoprotein (ox-LDL) via scavenger receptors (SRs) namely, CD36 and class A SR (SR-A), to form lipid-laden foam cells. These foam cells initiate an inflammatory cascade by releasing pro-inflammatory cytokines that accelerate lipoprotein retention and vascular inflammation [2].

Ample evidence in the literature report that macrophage apoptosis plays a dual role in atherosclerotic plaque development. In early fatty streak lesions, macrophage and foam cell apoptosis is accompanied with efficient efferocytosis, which limits lesion cellularity, suppresses inflammation, and plaque progression. Conversely, in advanced lesions inefficient efferocytosis leads to the accumulation of apoptotic macrophages, which favors the formation of a fibrous cap comprising of necrotic lipid-rich core and SMCs, thereby promoting inflammation, plaque disruption and thrombosis [3]. Therefore, novel therapeutic approaches which can reduce foam cell formation and enhance macrophage and foam cell apoptosis in the early stage of plaque development, need to be implemented for preventing the progression to advanced lesions.

Presently, synthetic anti-hyperlipidemic drugs like statins are widely used for treating cardiovascular disorders; however, these drugs have certain side effects [4]. Therefore, an alternative system of medicine like Ayurveda advocates the use of various medicinal plants, and one such plant is *Terminalia arjuna* (TA), which exhibits anti-inflammatory, anti-oxidant and hypolipidemic activities [5]. Consistent with this notion, our previous clinical trial data demonstrated that *T.arjuna* exhibits both anti-inflammatory and anti-atherosclerotic potential in patients with stable coronary artery disease, suggesting its cardio-protective role [6]. However, there is a dearth of information on anti-atherosclerotic therapeutic effect of the extracts obtained from these natural compounds, which can be employed for treating atherosclerosis by specifically targeting macrophages and foam cells in the initial stages of the lesion development. Further, the potential underlying mechanism implicated in macrophage and foam cell apoptosis at different stages of plaque development still remains elusive.

Recent evidence document that in atherosclerosis, free cholesterol (FC) trafficking to the endoplasmic reticulum (ER) membrane perturbs the integrity and function of ER membrane proteins, which results in the accumulation of unfolded or misfolded proteins and generates ER stress [7]. To combat stress and restore ER homeostasis, unfolded protein response (UPR) signaling pathway is activated, which initially exhibits a pro-survival role. ER stress is sensed by the three ER transmembrane receptors namely, inositol requiring enzyme 1 (IRE1), pancreatic ER kinase (PKR)-like ER kinase (PERK), and activating transcription factor 6 (ATF6). The primary function of these three signaling proteins is to alleviate the accumulation of unfolded/misfolded proteins in the ER by augmenting the selective expression of ER chaperones, suppressing mRNA translation and inhibiting protein synthesis, and by stimulating the transportation of misfolded proteins into the cytosol for the proteasomal degradation, thereby reducing the load of unfolded proteins and relieving ER stress [8]. However, under chronic ER stress conditions, UPR fails to resolve stress and switches the signaling branch from pro-survival to pro-apoptotic by triggering IRE1/ASK1 (apoptosis-signaling-kinase 1) -mediated activation of stress-kinase pathways including c-Jun N-terminal kinase (JNK) and p38 mitogen-activated protein kinase (MAPK), which in turn enhances the transcription of C/EBP homologous protein (CHOP) and X-box binding protein 1 (XBP1), subsequently leading to cell death [9]. CHOP is a pro-apoptotic transcription factor which mediates ER stress-induced cell death via the regulation of Bcl-2 family proteins [10]. Further, it has also been reported that p38MAPK phosphorylates XBP1, a transcription factor, which further cooperates with ATF6 to activate CHOP. Thus, both XBP1 and CHOP play a crucial role in ER-stress-induced apoptosis [11, 12].

Furthermore, our previous in vitro study (data unpublished) demonstrated that TA treatment led to a significant decline in dual-specificity phosphatase1 (DUSP1) expression levels in THP1 cells. It is well established that DUSP1, a phosphatase, preferentially dephosphorylates stress-activated kinases such as c-Jun N-terminal kinase (JNK) and p38 mitogen-activated protein kinase (MAPK), which are also the mediators of unfolded protein response (UPR)-induced apoptotic signaling cascade [13, 14]. Moreover, It has been postulated that ox-LDL promotes macrophage apoptosis by induction of the UPR pathway in ER-stressed macrophages [15]. Hence, we hypothesized that TA may promote apoptosis via directly modulating JNK and p38MAPK pathway in a DUSP1-dependent manner, and/or may also enhance ox-LDL induced ER stress-activated JNK and p38MAPK-mediated- apoptotic pathway. Therefore, the present study aimed to explore the pro-apoptotic effect of aqueous bark extract of TA (aqTAE) on macrophage and foam cells, and the underlying molecular mechanism associated with it.

Further, prior studies have demonstrated that polyherbal extracts exhibit antiatherogenic activity by inhibiting foam cell formation; however, there is a scarcity of data regarding the aqTAE’s effect on foam cell development. Hence, we also assessed the effect of aqueous bark extract of TA (aqTAE) on ox-LDL-induced THP1- derived foam cell formation. Collectively, these findings may provide novel mechanistic insight into the mode of action of TA, which may delay plaque progression in the early stage of atherosclerosis.

## Material and methods

### Preparation of aqueous extract of *T. arjuna* (aqTAE)

The bark of *T. Arjuna* (TA) was collected from a local Arjuna Tree, and was submitted in the herbarium of Panjab University, Chandigarh, where it was identified and authenticated by a certified botanist (voucher no. 20585). For the preparation of aqTAE, the bark was dried and coarsely powdered, and 100 g of the fine powder was dissolved in 1000 ml of distilled water. Further, the mixture was subjected to boiling with continuous stirring for 6 h at 100 °C, followed by its cooling at room temperature. After completion of the extraction process, the solvent was removed under reduced pressure using rota evaporator to obtain a deep reddish-brown lyophilized powder. Aliquots of aqTAE were stored in the dark, at − 20 °C for subsequent experiments.

### HPLC profiling of TA bark extract

The polyphenolic content in aqueous bark extract of *T. arjuna* was determined using an HPLC method. HPLC analysis was performed on a C18 column (300 mm * 3.9 mm ID, 5 μm, Supelco U.S.A), maintained at a temperature of 30 °C. Mobile phases used were a) 0.1% Formic acid in water b) 0.1% Formic acid in HPLC grade acetonitrile, and the flow rate was 1 ml/min. The chromatographic peaks of the analytes in the extract were identified and confirmed by comparing their retention time with those of the reference standards i.e., gallic acid (GA), ellagic acid (EA) and epigallocatechin gallate (EGCG), by a photodiode array detector (PDA) at an absorbance of 258 nm. The total polyphenolic content of aqTAE was determined spectrophotometrically by Folin-Ciocalteau assay [16]. A standard curve for EGCG was plotted, and the polyphenol content in our laboratory prepared extract of *T. arjuna* was extrapolated based on the standard curve and was expressed as mg/g of extract.

### Preparation of ox-LDL

Human LDL was isolated from the fresh plasma of healthy donors by the sequential ultra-centrifugation method, and the concentration of LDL was estimated by Qubit reagent (Molecular Probes, Invitrogen, USA) [17]. Further, oxidized LDL was prepared by incubating LDL fraction (5 mg/ml diluted in PBS) with 10 μM CuS0_4_ for 48 h at 37 °C. The extent of oxidative modified LDL was evaluated by examining relative electrophoretic mobility assay and by measuring thiobarbituric acid-reactive substances (TBARS) [18].

### Cell culture and treatment protocol

Human THP1 monocytes were cultured in RPMI-1640 media supplemented with 10% fetal bovine serum (FBS) (Sigma-Aldrich, USA), 200 mM L-Glutamine 10mM HEPES, 1 mM sodium pyruvate, 100 μg/ml streptomycin and 100 U/ml of penicillin, and 0.05 mM β-mercaptoethanol. The cells were maintained in a humidified atmosphere with 5% CO_2_ at 37 °C, and the passage range of 5–14 were used for further experiments. Thereafter, the cells at a density of 5*10^5^ /ml/well were seeded in six-well culture plates and differentiated into macrophages by stimulation with 100 ng/ml PMA (phorbol-12-myristate-13-acetate) for 24 h. Later, THP1 macrophages were treated with different doses (50–200 μg/mL) of TA extract for a maximum of 24 h, and a significant increase in apoptosis (~ 50%) was observed with 100 μg /mL concentration, therefore, this optimal dose of *T. arjuna* extract was chosen for subsequent experiments. Four different experimental conditions were assayed on THP1 macrophages: (1) Cells were incubated with ox-LDL (100 μg/ml) alone for 24 h to induce foam cell formation. (2) Cells were incubated with TA (100 μg/ml) alone for 24 h. (3) Cells were co-treated with ox-LDL and TA for 24 h. (4) Cells were treated with ox-LDL for 24 h, followed by TA for 24 h. The untreated macrophages were employed as a control group.

### Foam cell formation by oil red O (ORO) staining

Briefly, after treatment, the cells were rinsed with PBS, fixed with 10% phosphate-buffered formalin for 10 min, followed by their washing with PBS once. The fixed cells were stained with filtered 0.5% ORO solution (Sigma-Aldrich, USA) at 37 °C for 15 min in darkness, followed by destaining with 60% isopropanol for 15 s and washing thrice with PBS. Further, Oil red O-stained lipid-laden macrophages in different experimental groups were observed under light microscope (Olympus Corp., Tokyo, Japan).

### Flow cytometric analysis of CD36 surface expression

Briefly, the cells were harvested after the completion of respective treatments, and washed twice with PBS. Further, 2*10^5^ cells/well were stained with 200 μl PBS containing 20 μl of FITC-conjugated anti-human CD36 monoclonal antibody (Immunotech, Westbrook, USA) on ice for 40 min. After washing twice with ice-cold PBS, the samples were analyzed by FacsDIVA software (Becton-Dickinson Franklin Lakes, NJ, USA).

### Detection of apoptosis by flow cytometry

After the completion of the treatment protocol, cells were harvested, washed with PBS, and stained with 4 μl of 10 μM Mitotracker Red suspended in 1 ml RPMI-1640 medium, for 30 min at 37 °C in a humidified 5% CO_2_ incubator. Subsequently, the cells were washed twice with PBS, and 5 μl of Alexa Fluor 488-labelled Annexin V (Invitrogen, CA, USA) in 100 μl of 1X Annexin binding buffer was added. The samples were then incubated on ice for 15 min in the dark. The relative number of cells that were annexin V- positive (apoptotic) and mitotracker red-positive (viable) were determined using FACS Aria II flow cytometer (Becton Dickinson, Franklin Lakes, NJ, USA).

### Real-time PCR

The cultured cells were harvested and suspended in 1 ml of TRIZOL reagent (Life Technologies), and 1 μg of total RNA was reverse transcribed to cDNA template using cDNA synthesis kit (Thermo Fisher Scientific, MA, USA). The mRNA expression of *XBP1* and *CHOP* genes was determined by quantitative real-time PCR (qRT-PCR) using SYBR Green chemistry (Applied Biosystems, Foster City, CA). The sequences of the human-specific primers used were as follows: **XBP1, F:** 5′-CTGAGTCCGCAGCAGGTG-3′, **R:** 5′-GGCTGGTAAGGAACTGGGTC-3′; **CHOP**, **F:** 5′-TTCTCTGGCTTGGCTGACTG-3′, **R:** 5′-TCCTCCTCTTCCTCCTGAGC-3′; **β-actin**, **F:** 5′-GGCACCCAGCACAATGAAG-3′, **R:** 5′-ACTCGTCATACTCCTGCTTG-3′. For XBP1, the PCR cycles consisted of initial denaturation at 95 °C for 10 min; followed by 35 cycles of denaturation at 95 °C for 15 s; annealing at 56 °C for 25 s; extension at 72 °C for 30 s. To amplify CHOP mRNA, PCR was performed with an initial denaturation at 95 °C for 10 min, followed by 35 cycles of denaturation at 95 °C for 15 s; annealing at 60 °C for 25 s; extension at 72 °C for 25 s. The relative mRNA levels were normalized to human β-actin mRNA expression, and the values are expressed in fold change, as evaluated by the 2^-ΔΔCT^ method [19].

### Immunoblotting

Cultured cells were harvested and lysed in RIPA buffer containing a protease inhibitor cocktail (Sigma-Aldrich, USA). Cell lysates with equal protein concentration were subjected to western blot with primary antibodies against CD36 (Abcam, Cambridge, MA, USA), and CHOP (Biorbyt, Cambridge, MA, USA) followed by incubation with respective HRP-conjugated secondary antibodies for 1 h. Immune reactive bands were detected by enhanced chemiluminescence system (Fluorchem M, Protein simple). Densitometric analysis was performed by Image J software (1.47 v), and the levels of target proteins were normalized to β-actin.

### Statistical analysis

Data are expressed in mean ± SD. The significance of mean values of different parameters between the treatment groups was analyzed using one-way analysis of variances (ANOVA) followed by post-hoc Tukey test. *P* values < 0.05 were considered to be statistically significant. All the statistical analysis was performed using Graph Pad Prism (version 6, California, USA).

## Results

### Phytochemical analysis of TA bark extract

**Fig. 1:**
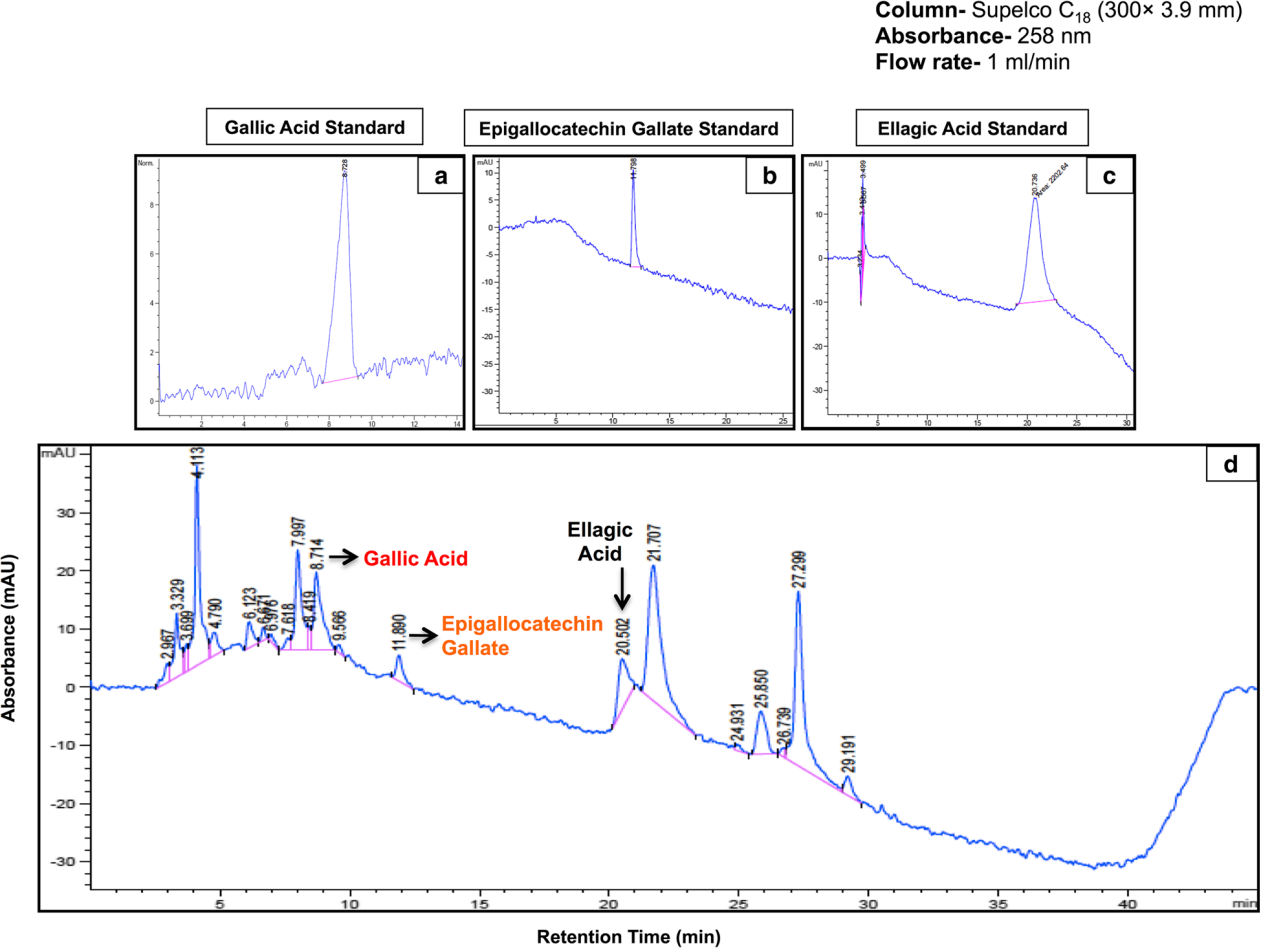
(**a-c**) Reference chromatograms (**d**) Representative HPLC-chromatogram indicating the presence of gallic acid, epigallocatechin galate and ellagic acid along with other compounds in aqueous bark extract of *T. arjuna*

The percentage yield of the powdered extract was found to be approximately 18.3%, and the total phenolic content present was almost 170 mg/g of dried aqueous bark extract of TA. Further, the peaks of compounds, namely gallic acid, epigallocatechin gallate and ellagic acid in the extract were characterized by comparing with the standard chromatograms **(**Fig. 1)**.**

### *T. arjuna* bark extract prevents ox-LDL-induced foam cell formation in THP1 cells

**Fig. 2:**
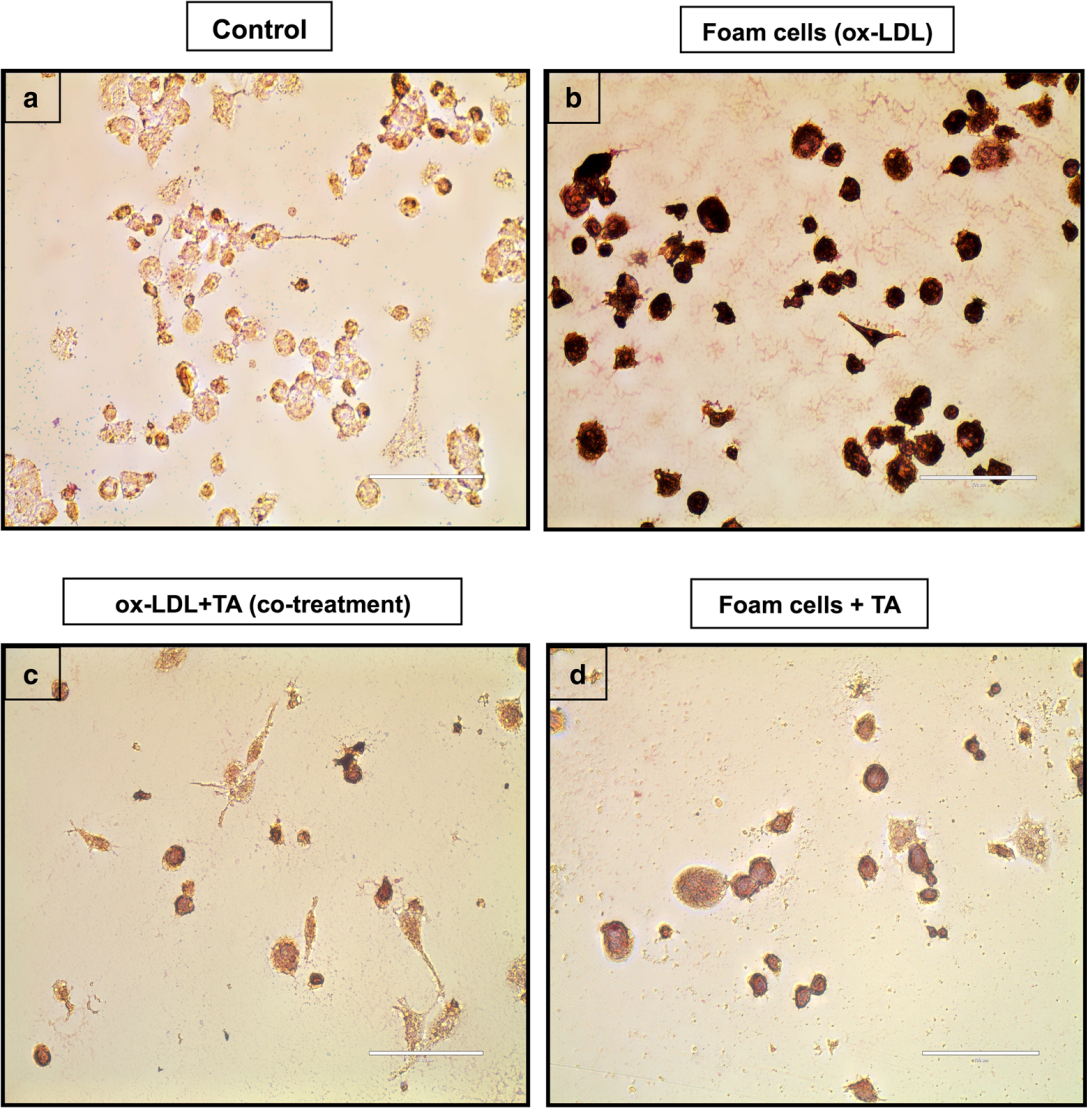
Effect of aqTAE on macrophage-derived foam cell formation. Representative microscopic images of Oil Red O-stained lipid droplets (40x magnification) in different experimental groups (**a**) Control (untreated macophages); (**b**) Foam cells (ox-LDL); (**c**) ox-LDL+TA (co-treatment); and (**d**) Foam cells + TA

**Fig. 3:**
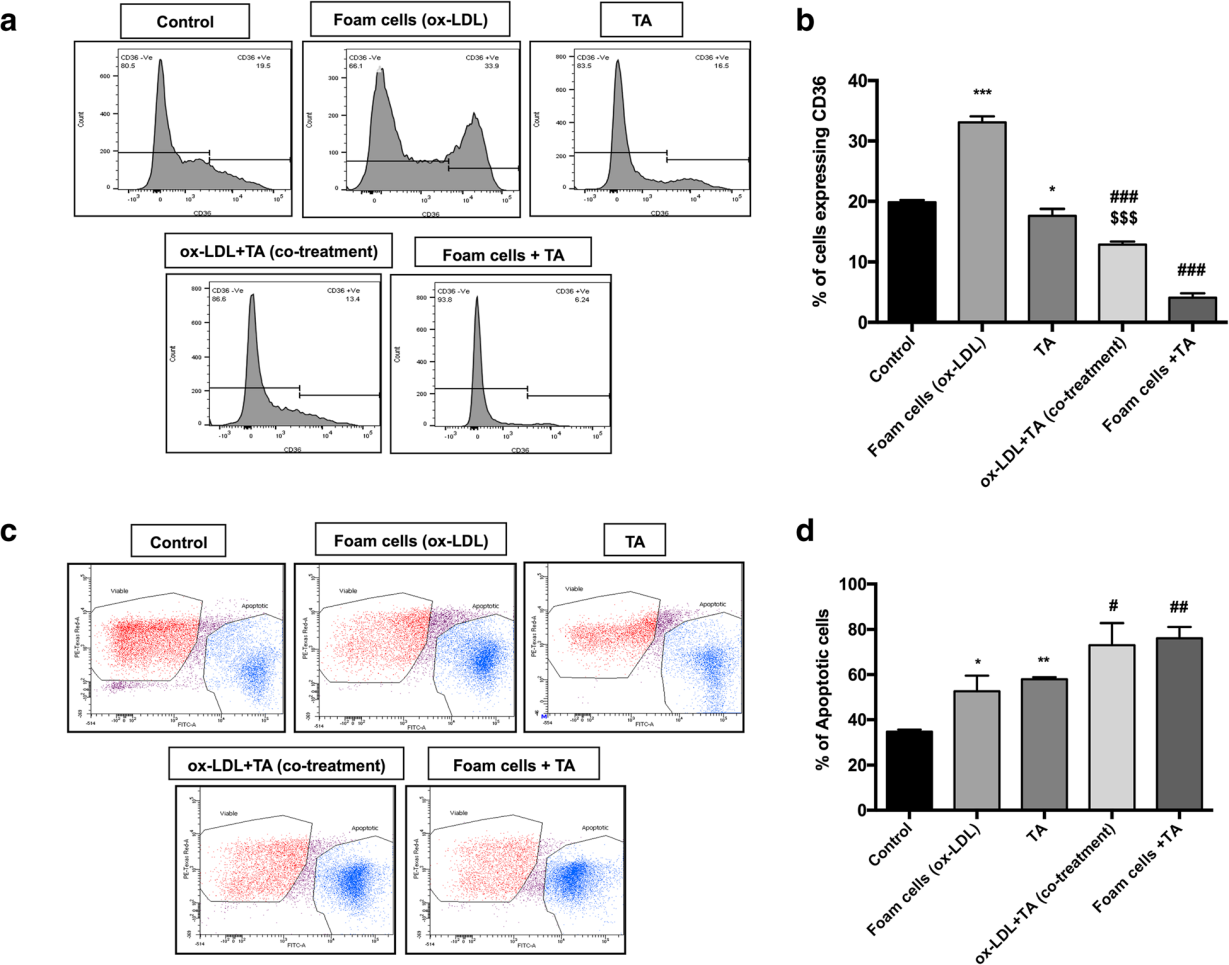
Effect of aqTAE on CD36 surface expression on macrophages and foam cells (top panel). (**a**) Representative histograms of CD36 expression by FACS and (**b**) bar diagram displaying % of cells expressing CD36 in the different experimental groups. Effect of aqTAE on macrophage and foam cell apoptosis (bottom panel). (**c**) The dot plots representing viable and apoptotic cell populations using Annexin V-FITC/Mitotracker Red staining by flow cytometry and (**d**) the mean percentage of apoptotic cells in all the experimental groups. Data expressed as mean ± S.D. (*n* = 3). (* = vs. control) (# = vs. ox-LDL alone) ($ = vs. TA alone); ^*^p < 0.05, ^**^p < 0.01, ^***^p < 0.001, ^**#**^*p* < 0.05, ^**##**^*p* < 0.01, ^**###**^*p* < 0.01, ^**$$$**^*p* < 0.001

**Fig. 4:**
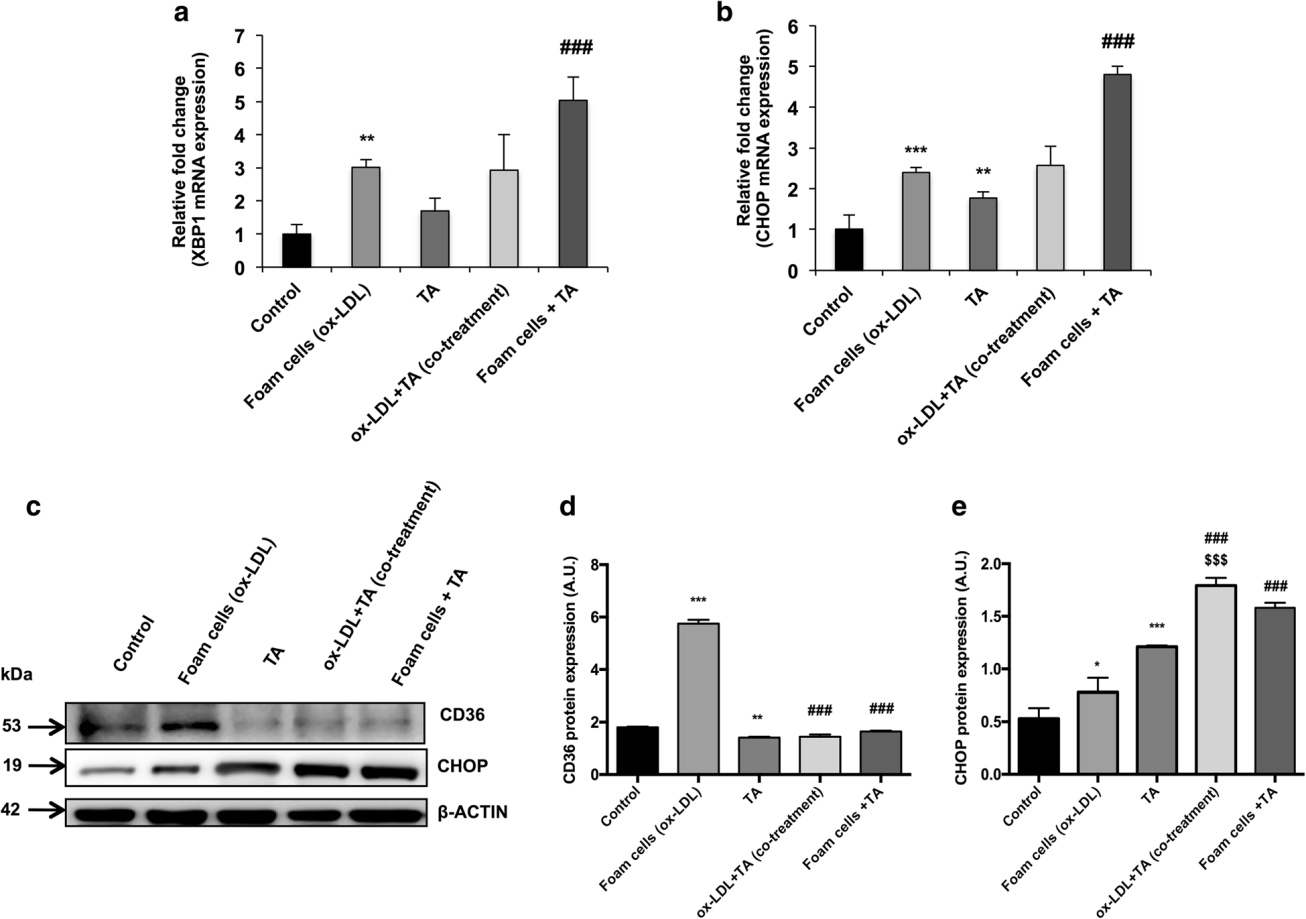
Effect of aqTAE on protein expression levels of CD36 and UPR-related markers in macrophages and foam cells. (**a**) and (**b**) Depicts mRNA levels of *XBP1 and CHOP* in the different groups. (**c-e**) Representative blots and quantification of protein levels of CD36 and CHOP normalized to the β-actin levels. Data expressed as mean ± S.D. of at least 3 independent experiments. ^*^p< 0.05, ^**^p < 0.01, ^***^p < 0.001 vs. control group, ^**###**^*p* < 0.001 vs. ox-LDL group, ^$$^^$^*p* < 0.05 vs. TA alone

To assess the effect of aqTAE on foam cell formation; we performed ORO staining, and observed that the ox-LDL treatment alone markedly increased the percentage of foam cells, while co-treatment with ox-LDL and *T.arjuna* bark extract led to a substantial decline in Oil red O-positive lipid-laden macrophages, indicating reduced foam cell formation (Fig. 2). Similarly, flow cytometry and western blot analysis revealed that CD36 expression was significantly upregulated by ox-LDL alone as compared to the macrophages (control) (*p* < 0.05), while, addition of TA along with ox-LDL significantly downregulated CD36 expression, as compared to the TA alone and ox-LDL- treated alone group, respectively (*p* < 0.05) (Figs. 3a and b, 4c and d).

### *T. arjuna* bark extract induces macrophage and foam cell apoptosis

To determine whether *T.arjuna* induces apoptosis of macrophages and foam cells, the percentage of apoptotic cells was examined using Annexin/Mitotracker staining by flow cytometry. We observed that treatment with ox-LDL and TA, respectively, caused a significant increase in the percentage of apoptotic cells, as compared to the control group (*p* < 0.05 for both). Moreover, when the macrophages were co-incubated with ox-LDL and TA, a significant increase in the apoptosis was observed, as compared to the ox-LDL alone (*p* < 0.05), while the apoptotic levels were found to be insignificantly higher, when compared to TA alone. Similarly, aqTAE treatment also led to a significant increase in the foam cell apoptosis, as compared to the group treated with ox-LDL alone (*p* < 0.05) **(**Fig. 3c and d**)**. These observations were further confirmed by ORO staining, FACS and western blot analysis of CD36 expression, which revealed that both [ox-LDL + TA (co-treatment)] group and aqTAE-treated foam cells demonstrated a significant decline in ORO positive cells, accompanied with a decrease in CD36 expression, as compared to their respective controls i.e. TA alone and ox-LDL alone (*p* < 0.05 for each) **(**Figs. 2, 3a and b, 4c and d**)**. In addition, TA-treated macrophages also displayed a significant downregulation in CD36 expression, relative to the untreated group (*p* < 0.05) **(**Figs. 3a and b, 4c and d**)**.

### *T. arjuna* bark extract modulates ER stress-induced UPR pathway in THP1 cells

To explore whether TA- induced macrophage and foam cell apoptosis is mediated via UPR pathway, we examined the expression of ER-stress markers i.e., XBP1 and CHOP by real-time qPCR and western blot analysis. Our results showed that both *XBP1* and *CHOP* mRNA expression levels were higher, though insignificant in ox-LDL + TA (co-treated)-group, when compared to the groups treated with either TA-alone or ox-LDL-alone. However, the mRNA levels of both the genes were significantly upregulated in aqTAE-treated foam cells as compared to the group treated with ox-LDL alone (*p* < 0.05 for each). Further, CHOP protein expression was found to be significantly augmented in the macrophages when co-treated with ox-LDL and TA, as compared to their respective controls i.e. TA alone and ox-LDL alone (*p* < 0.05 for each), and a similar observation was noted in TA-treated foam cells, when compared to the ox-LDL treatment alone (*p* < 0.05). Further, macrophages when stimulated independently with ox-LDL and TA extract displayed significantly increased expression of XBP1 and CHOP at both mRNA and protein levels, relative to the untreated group (*p* < 0.05 for each) **(**Fig. 4**)**.

## Discussion

In early atherosclerotic lesions, macrophage and foam cell apoptosis plays a key role in limiting lesion cellularity and suppressing atherosclerotic inflammation that eventually results in plaque regression [20]. Further, unfolded protein response (UPR) pathway exhibits both pro-survival and pro-death functions, and thus, specifically targeting this response by suppressing the adaptive arm or enhancing pro-death signaling may prove clinically beneficial in the treatment of atherosclerosis. Therefore, therapeutic approaches to modulate macrophage-derived foam cell formation, and to promote macrophage and foam cell apoptosis by modulating UPR-mediated apoptotic pathway in early stages of atherosclerosis may emerge as a novel and efficient strategy for the prevention and treatment of cardiovascular diseases. In the present study, we demonstrated that *T. arjuna* bark extract inhibits CD36-mediated ox-LDL uptake by macrophages, thereby preventing the ox-LDL accumulation and subsequent foam cell formation. Additionally, it was also observed that aqTAE significantly increased macrophage and foam cell apoptosis by modulating DUSP1-mediated activation of JNK/p38MAPK pathway, thus coupling MAPK to ER stress signaling, and further promoting apoptosis through the activation of UPR-related proteins including CHOP and XBP1.

In recent years, traditional Ayurvedic medicine “***Terminalia Arjuna***” known for its beneficial hypolipidemic and cardioprotective actions has gained much attention and is being widely used for the treatment of cardiovascular diseases. Our previous in vitro and in vivo studies have also demonstrated the anti-inflammatory, anti-atherosclerotic, and immunomodulatory effects of *T. arjuna* [6]. We, therefore, sought to study the effect of aqueous bark extract of *T. arjuna* on macrophage foam cell formation, the hallmark of early atherosclerotic lesions. Our results showed that ox-LDL treatment alone promoted macrophage-derived foam cell formation as indicated by a remarkable increase in the CD36 expression and Oil Red O (ORO) staining. However, on co-treatment with ox-LDL and TA bark extract, there was a marked decline in CD36 expression and ORO positive cells. These findings imply that *T. arjuna* bark extract alleviates foam cell formation by suppressing ox-LDL-induced CD36 expression, concomitantly inhibiting CD36-mediated ox-LDL uptake by macrophages to prevent the formation of lipid-laden foam cells. Another possible reason could be due to increased macrophage apoptosis in [ox-LDL + TA (co-treatment)] group, which may have further attenuated macrophage-derived foam cell formation, and this observation is further supported by the increased percentage of apoptotic cells in TA alone-treated macrophages. Taken together, to the best of our knowledge, our study is the first report to demonstrate the inhibitory effect of TA bark extract on foam cell formation.

**Fig. 5:**
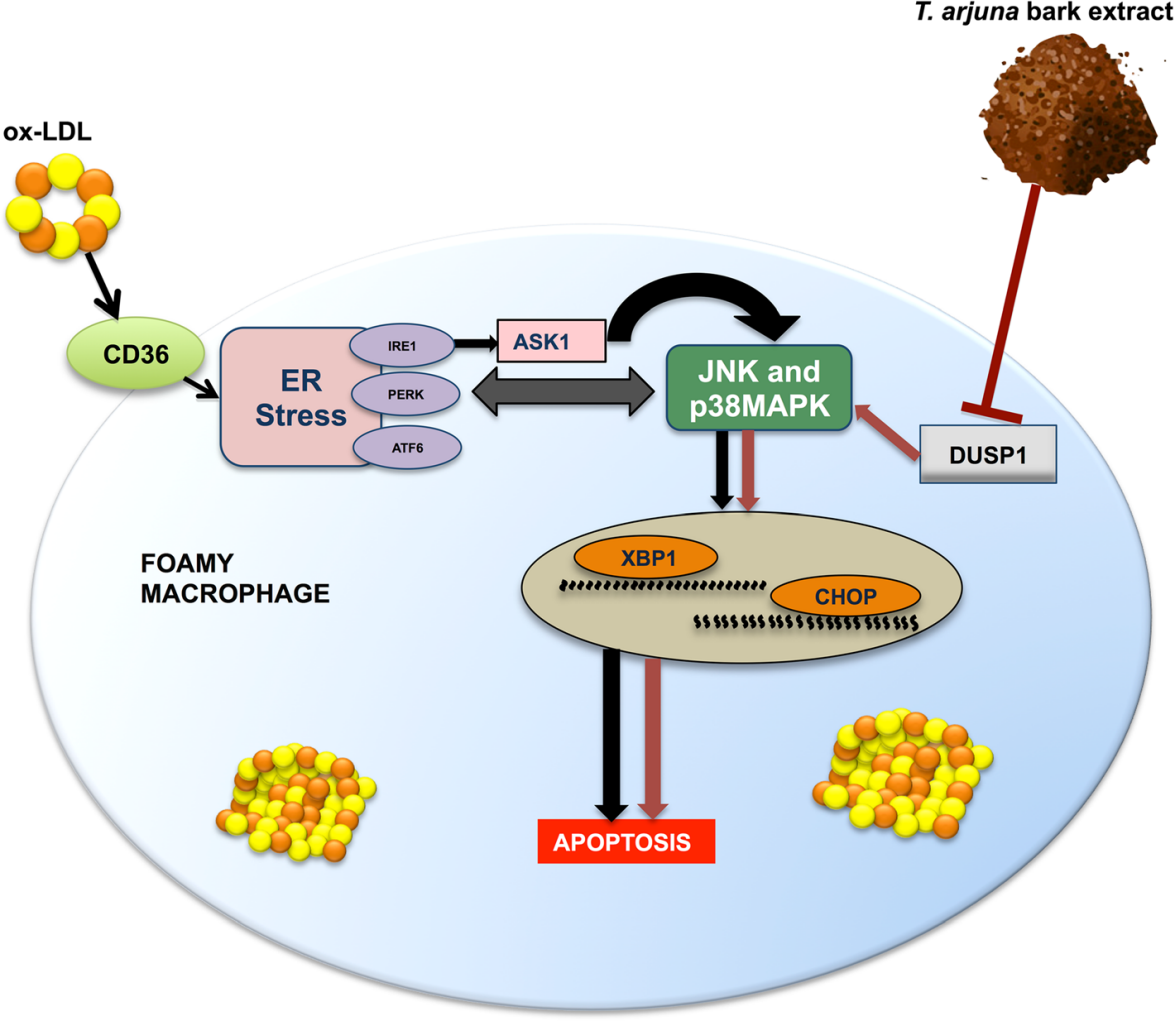
Proposed model depicting the underlying molecular mechanism by which *aqueous bark extract of T. arjuna* exerts pro-apoptotic effect on macrophage and foam cells. TA promotes macrophage and foam cell apoptosis by directly modulating JNK, p38MAPK-CHOP pathway in a DUSP1-dependent manner (indicated by red coloured lines), and may also augment ox-LDL induced UPR-mediated activation of JNK and p38MAPK-CHOP cascade (black coloured lines), suggesting a possible interplay between the ER stress- and TA-mediated MAPK signaling (denoted by bidirectional arrow)

It is well established that ox-LDL triggers apoptosis in ER-stressed macrophages by activating UPR-induced CHOP pathway [20]. In view of the same, our results demonstrate that ox-LDL treatment alone significantly enhanced macrophage apoptosis, as evidenced by increased Annexin V positive staining, which was accompanied with augmented expression levels of ER stress signature genes including XBP1 and CHOP. These observations indicate that ox-LDL induces ERS (endoplasmic reticulum stress), which may subsequently trigger UPR-mediated IRE1/ASK1-dependent activation of JNK and p38MAPK signaling pathway, thereby further leading to macrophage apoptosis via the upregulation of XBP1 and CHOP. Similarly, when the macrophages were exposed to aqTAE alone, there was a significant increase in the percentage of apoptotic cells, followed by elevated levels of ER stress markers. Our previous in vitro study in THP1 macrophages showed that TA suppresses DUSP1, a MAP kinase phosphatase 1, which is known to negatively regulate JNK/P38MAPK signaling cascade. Therefore, it is conceivable that TA promotes macrophage apoptosis by attenuating DUSP1 levels, which may in turn cause the prolonged phosphorylation/activation of JNK/P38MAPK pathway, consequently leading to XBP1/CHOP-mediated apoptosis, as both JNK and p38MAPK are well-known regulators of XBP1 and CHOP activation [11, 21, 22]. Further, it was interesting to note that the macrophages co-treated with ox-LDL and *T. arjuna* bark extract, and aqTAE-treated foam cells displayed maximally elevated levels of apoptosis and UPR pathway components, as compared to THP1 macrophages treated either with TA or ox-LDL alone. These findings depict that treatment with TA may have resulted in further augmentation of ox-LDL-induced ER stress-activated JNK and p38MAPK pathway in a DUSP1-dependent manner, which subsequently accelerated foam cell apoptosis via upregulation of XBP1 and CHOP, indicating a possible cross-talk between endoplasmic reticulum (ER) stress- and TA-mediated JNK/p38MAPK signaling pathway (Fig. 5). In view of the above observations, our study is the first to report the pro-apoptotic effect of TA bark extract on foam cells and macrophages, highlighting the cardio-protective potential of TA, which may prove beneficial in the early stage of atherosclerosis.

In conclusion, TA inhibits ox-LDL-induced CD36 expression, which further limits ox-LDL uptake by the macrophages, thereby impeding macrophage-derived foam cell formation. Additionally, aqTAE promotes macrophage and foam cell apoptosis through DUSP1-mediated activation of JNK/p38MAPK signaling, which is coupled to the UPR-CHOP - apoptotic pathway in ER-stressed macrophages. These findings suggest a cross-talk between TA- and UPR- mediated MAPK signaling, thereby providing novel insights into the anti-atherosclerotic mechanism of action of *T. arjuna*. Therefore, our data reveals that TA bark extract may act as an an effective anti-atherogenic phytochemical, which could be ascribed to its bioactive phytoconstituents, underscoring its therapeutic potential for the treatment of atherosclerosis.

## Data Availability

The datasets analyzed during the current study are included in the manuscript.

## Abbreviations

aqTAE: aqueous bark extract of *Terminalia arjuna*
CHOP: CAAT enhancer binding protein (C/EBP) homology protein
DUSP1: Dual-specificity phosphatase1
ER: Endoplasmic reticulum
ORO: Oil Red O
TA: *Terminalia Arjuna*
XBP1: X-box binding protein 1
